# Concurrent posterior vault expansion and extradural Chiari decompression in syndromic and non-syndromic craniosynostosis: a case series

**DOI:** 10.1007/s00381-025-07004-y

**Published:** 2026-01-26

**Authors:** Isabel A. Ryan, Ashley E. Chang, Philip D. Tolley, Jordan W. Swanson, Scott P. Bartlett, Shih-shan Lang, Gregory G. Heuer, Jesse A. Taylor

**Affiliations:** 1 Division of Plastic, Reconstructive, and Oral Surgery, Children’s Hospital of Philadelphia, Philadelphia, PA USA; 2 Division of Neurosurgery, Children’s Hospital of Philadelphia, Philadelphia, PA USA; 3 Children’s Hospital of Philadelphia, Perelman School of Medicine at the University of Pennsylvania, 3401 Civic Center Blvd, Philadelphia, PA USA

**Keywords:** Chiari malformation, Craniosynostosis, Posterior vault distraction osteogenesis, Posterior vault reconstruction

## Abstract

**Purpose:**

Chiari malformation (CM) may occur in patients with craniosynostosis, and the presence of both in anatomically similar locations allows for simultaneous treatment. In this study, we analyze concurrent posterior vault distraction osteogenesis (PVDO) or posterior vault reconstruction (PVR) with extradural Chiari decompression for the management of suspected increased intracranial pressure (ICP) in patients with syndromic and atypical non-syndromic craniosynostosis.

**Methods:**

Patients who underwent concurrent PVDO/PVR and extradural Chiari decompression at a single institution from 2008 to 2024 were included. Of interest were patient/caregiver-reported symptomatology and radiographic findings related to CM reported by a board-certified neuroradiologist. Descriptive, Chi-square, and Fisher’s exact tests were utilized for analysis.

**Results:**

Eight patients met the inclusion criteria. Fifty percent (*n* = 4) had syndromic craniosynostosis and 50% (*n* = 4) had non-syndromic craniosynostosis; half (*n* = 4) underwent PVDO, and half (*n* = 4) underwent PVR. One patient (12.5%) had a post-operative complication (distractor infection), with no long-term sequelae. One hundred percent (*n* = 8) had resolution of clinical symptoms at the first follow-up, and 85.7% (*n* = 6/7) at the last follow-up; 50.0% (*n* = 4) of patients had improvement, and 50.0% (*n* = 4) had a stable presentation of CM on post-operative imaging. While most patients with radiologic improvement in CM were in the PVDO group compared to PVR (75% (*n* = 3/4) vs. 25% (*n* = 1/4), *p* = 0.5), this study was underpowered to detect a significant difference between techniques.

**Conclusions:**

Posterior vault expansion with extradural Chiari decompression in patients with concomitant suspected increased ICP and craniosynostosis appears safe and relatively effective at extended follow-up. Additional study of its long-term effects, patient selection, and comparison of PVDO vs PVR is warranted.

## Introduction

Chiari malformation (CM), or descent of the cerebellar tonsils below the foramen magnum, may occur in combination with craniosynostosis, either as a direct result of increased intracranial pressure or as an unrelated pathology [1–4]. CM may also result in part due to overcrowding of the posterior fossa secondary to underdevelopment of the occipital bone and decreased intracranial volume, which can occur in various forms of craniosynostosis (CS) [2]. Throughout childhood, patients with symptomatic CM or concern for increased intracranial pressure (ICP) and complex craniosynostosis may undergo intra- or extra-dural Chiari decompression to address symptoms of CM [4–7] or posterior vault surgery such as single-stage posterior vault reconstruction (PVR) or posterior vault distraction osteogenesis (PVDO) to address the sequelae of craniosynostosis including calvarial dysmorphology, decreased intracranial volume, and increased ICP [8–11].

In select patients, the benefits of performing these operations simultaneously may outweigh the additional risk from additive surgery. Cinalli et al. outlined a concurrent occipital remodeling and suboccipital Chiari decompression procedure for severe craniosynostosis and chronic tonsillar herniation, showing that Chiari decompression at the time of posterior vault surgery is feasible and relatively safe [12]. Similarly, Park et al. noted resolution of cerebellar tonsillar herniation after complete PVDO to the level of the foramen magnum in patients with craniosynostosis and Chiari I malformation [13]. In this study, we compare symptoms and radiographic appearance of CM pre- and post-operation in those patients in whom we performed concurrent PVDO/PVR and extradural Chiari decompression.

## Methods

### Study design

Following Institutional Review Board approval, retrospective review was used to identify patients who underwent concurrent PVDO/PVR and Chiari decompression at our institution from 2008–2024. Patients who had a diagnosis of CM and craniosynostosis, as well as at least one pre-operative and post-operative scan (MRI or CT scan) and post-operative visit were included. Patient demographic, operative, and follow-up data were collected. In patients undergoing PVDO, distraction distance was measured using lateral radiographs after beginning consolidation. CM diagnosis and herniation degree were based on CT or MRI findings dictated by a board-certified, pediatric neuroradiologist. Pre-operative imaging closest to the time of surgery and most recent post-operative imaging were analyzed. In one patient with multiple decompressions, post-operative imaging before subsequent decompressions was considered. Change in CM was based on radiologist comments detailing the degree of tonsillar herniation or noting improvement, worsening, or stability of tonsillar herniation/ectopia. Diagnosis of shunt-dependent hydrocephalus (SDH) was based on pre-operative clinic and operative notes. Clinical symptomatology was assessed at the first and last post-operative visit for all patients, with symptoms including new headaches, head-banging, vision changes, and feeding and sleeping issues (central sleep apnea) reported by children or parents and felt to be attributable to increased intracranial pressure (ICP) or CM by the attending neurosurgeon.

### Surgical technique

Decision to undergo PVDO versus PVR is determined on an individual basis, considering pre-operative cranial morphology and the need for intracranial volume expansion.

#### PVDO

**Fig. 1:**
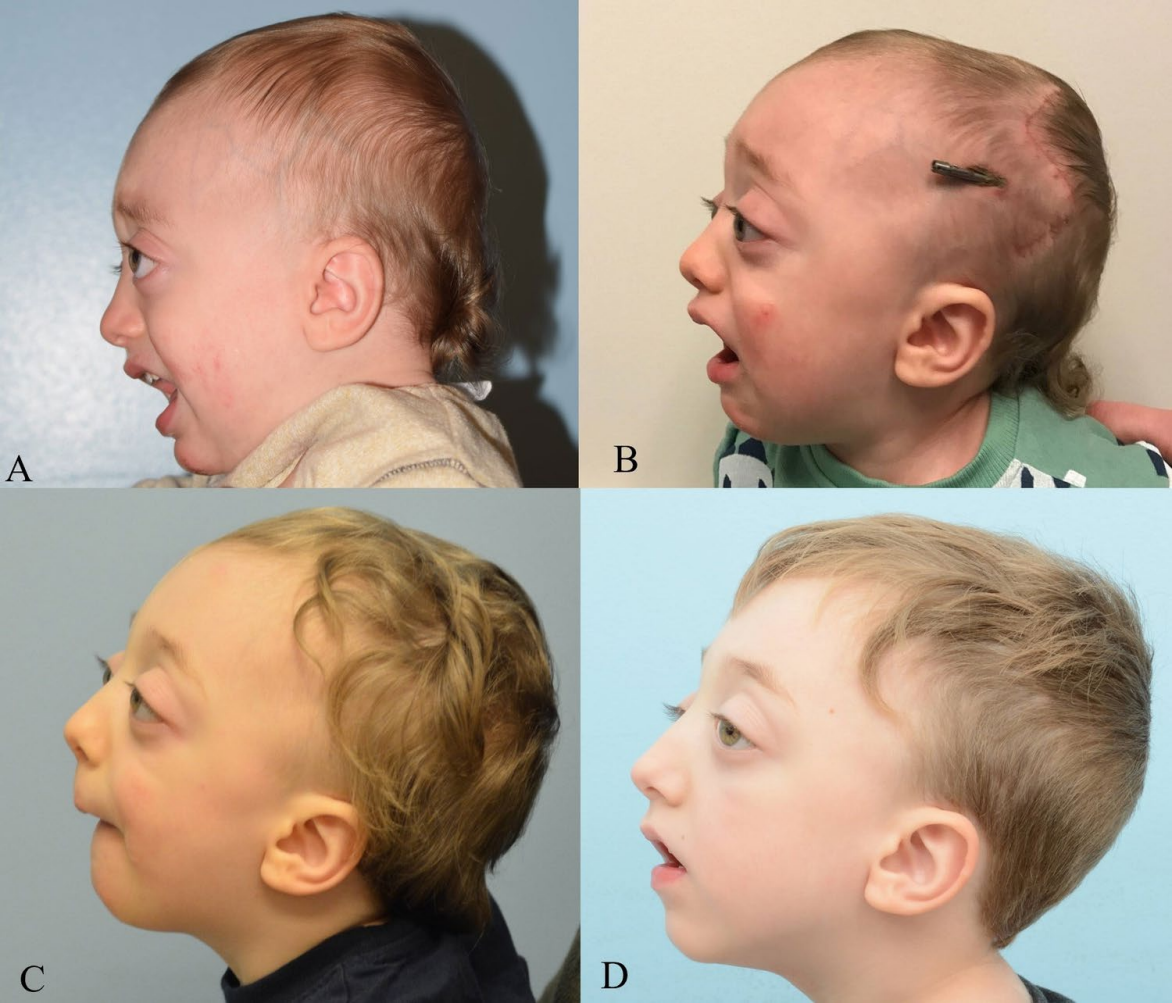
A patient with Crouzon syndrome and Chiari I malformation presented at 14 months of age with mild supraorbital retrusion and exorbitism (**A**). He was noted to have enlarged ventricles, aqueductal stenosis, and acquired Chiari malformation. At 15 months, he underwent concurrent posterior vault distraction osteogenesis and extradural Chiari decompression. He is seen here 1-month post-operation (**B**). After 1 month of active distraction, he underwent distractor removal and showed improvement in posterior vault volume pictured here 9 months post-removal (**C**). At 3 years post-operation, he continued to be symptom free and has not undergone additional craniofacial operations (**D**)

**Fig. 2:**
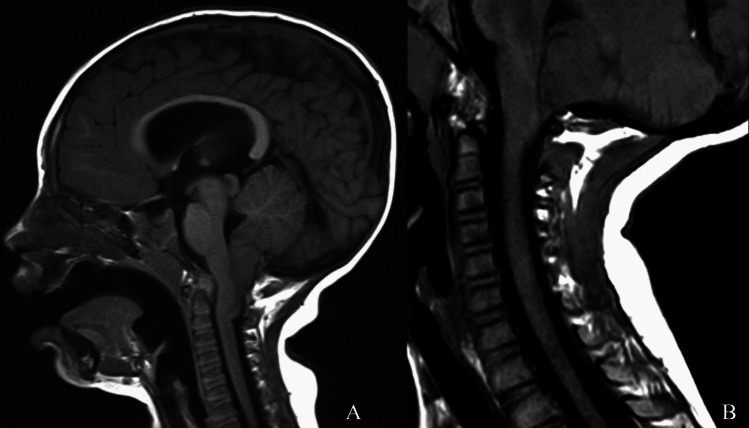
Pre-operative MRI brain (**A**) and post-operative MRI C-Spine (**B**) in a patient with Crouzon syndrome and Chiari I who underwent posterior vault distraction osteogenesis with concurrent extradural Chiari decompression. Pre-operative tonsillar herniation was 13 mm. At 29 months post-operatively, tonsillar herniation was improved at 6 mm

The technical details of PVDO performed at our institution have been described previously [8, 14]. Concurrent Chiari decompression represents an adjunct to this, requiring increased coordination between the plastic surgeon and neurosurgeon. The patient is first positioned prone, and a coronal incision is then made to allow for exposure of the posterior and middle vault. Subgaleal undermining of the posterior vault and middle vault is then carried out, transitioning to the subperiosteal plane in the low occipital vault to the cranial base. Working together, the neurosurgical and craniofacial teams perform middle and posterior vault osteotomies followed by extradural Chiari decompression, essentially removing the central bony segment below the transversely oriented occipital osteotomy, carrying the dissection down to the foramen magnum. This includes occipital barrel stave osteotomies that are subsequently greenstick outfractured to allow for a smooth transition between the cranial base and the transport segment. Colinear posterior vault distractors are then placed in the low temporal region of the calvarium to allow for anterior–posterior expansion with a slight inferior vector. After a latency period (2–5 days), distraction begins at a rate of 1 mm/day. Our protocol is to consolidate for approximately 2 months before removing the distraction devices. A patient example is detailed in Figs. 1 and 2.

#### PVR

Our technique for posterior vault reconstruction has also been well-documented, and the following is a brief description of it [15]. After exposure via a coronal incision, middle and posterior craniectomy is performed. This gives excellent exposure for subsequent extradural Chiari decompression. After bony expansion and fixation, particulate cranial bone graft is used to fill in bony gaps [16]. Our practice is to close over a suction drain for a limited time after surgery.

### Volumetric analysis

3D craniometric analyses were performed on pre-operative and most recent post-operative high-resolution CT scans. 3D reconstructions were created using Mimics (Version 26.0, Materialise, Leuven, Belgium). Anatomic landmarks including the sella, nasion, basion, euryon, opisthion, and frontozygomatic sutures were marked. Volumetric analyses were based on previously described methodologies [17, 18]. Intracranial space was isolated using 3-Matic (Version 15.0, Materialise, Leuven, Belgium) and imported into Mimics to allow for the calculation of the total intracranial volume. Two coronal planes based on the sella and basion were used to segment the intracranial volume into anterior, middle, and posterior volumes. The volume of each segment and the ratio of each segment as compared to the total intracranial volume was then calculated.

### Statistical analysis

Descriptive statistics were used to analyze demographic data. Categorical variables were analyzed using chi-square and Fisher’s exact tests based on data-specific parameters. Paired *t*-tests and Wilcoxon signed-rank tests were used to analyze volumetric changes as appropriate. Mann-Whitney *U* tests and Student’s *t*-tests were utilized to analyze volumetric change between expansion techniques. Power analyses were conducted to determine the effect size needed to detect a significant difference among certain variables. Analyses were performed on JASP (Version 0.18.1, JASP Team, 2023). Statistical significance was defined as *p* < 0.05.

## Results

### Patient characteristics (Tables 1 and 2)

**Table 1:** Demographic, peri-operative, and post-operative details of 8 patients who underwent concurrent posterior vault distraction osteogenesis or posterior vault reconstruction and Chiari decompression. PVDO, posterior vault distraction osteogenesis; PVR, posterior vault reconstruction; FOA, fronto-orbital advancement; SDH, shunt dependent hydrocephalus

Patient	Age at surgery (years)	Sex	Craniosynostosis diagnosis	Chiari type	Degree of tonsillar herniation (mm)	Syrinx?	Prior CF Procedures	Concurrent procedure	Length of stay	Complications	Chiari on post-operative imaging?	Syrinx on post-operative imaging?	SDH status	Symptoms at first follow-up?	Symptoms at last follow-up?	Subsequent procedures
1	2.1	F	Crouzon	I	10	No	None	PVDO	5	None	Improved	No	Stable, SDH	Improved	Improved	Monobloc
2	4.1	M	Multi-suture (CYP26B1)	I	N/A	Yes	PVDO, FOA	PVR	4	None	Stable	Yes, worsened	Stable, SDH	Improved	Improved	Monobloc
3	1.3	M	Crouzon	I	13	No	None	PVDO	5	None	Improved (6 mm)	No	Stable, no SDH	Improved	Improved	None
4	3.8	M	Multi-suture (unknown)	I	16	No	PVDO, Craniectomy, FOA	PVDO	4	None	Improved (8 mm)	No	Stable, no SDH	Improved	Improved	FOA
5	0.8	F	Acquired multi-suture	II	10	Yes	Craniectomy, Chiari Decompression	PVR	5	None	Stable	Yes, stable	Stable, SDH	Improved	N/A	None
6	9.0	M	Sagittal	I	8	Yes	PVR	PVR	8	None	Improved	No, resolved	Stable, no SDH	Improved	Improved	None
7	17.8	M	Acquired multi-suture	I	7.5	No	PVR, FOA	PVR	9	None	Stable	No	Stable, SDH	Improved	Improved	Cranioplasty
8	5.5	F	Sagittal	I	20	Yes	Chiari Decompression	PVDO	9	Distractor Infection	Stable	Yes, worsened	Stable, SDH	Improved	Symptom recurrence	Chiari Decompression × 2

**Table 2:** Patient characteristics of eight patients who underwent concurrent posterior vault reconstruction or posterior vault distraction osteogenesis. CF: craniofacial surgery. PVR: posterior vault reconstruction. PVDO: posterior vault distraction osteogenesis. SDH: shunt dependent hydrocephalus

	*N* (%) or median (IQR)
**N**	8
**Age at presentation (months)**	0.4 (1.8)
**Sex**	
F	3 (37.5%)
M	5 (62.5%)
**Race**	
Caucasian or White	7 (87.5%)
African American or Black	1 (12.5%)
**Ethnicity**	
Not Hispanic or Latino	8 (100%)
**Non-genetic craniosynostosis**	4 (50.0%)
Sagittal	2 (25.0%)
Acquired multi-suture	2 (25.0%)
**Genetic craniosynostosis**	4 (50.0%)
Crouzon	2 (25%)
Multi-suture	2 (25.0%)
CYP26B1	1 (12.5%)
Unknown	1 (12.5%)
**Chiari malformation**	8 (100%)
Chiari I	7 (87.5%)
Chiari II	1 (12.5%)
**Tonsillar herniation (mm)**	10.0 (5.5)
**Syrinx on pre-operative imaging**	4 (50.0%)
**SDH pre-operatively**	5 (62.5%)
**Prior CF surgery**	6 (75%)
PVDO	2 (25%)
PVR	2 (25%)
Fronto-orbital advancement	3 (37.5%)
Craniectomy	2 (25%)
Chiari decompression	2 (25%)
**Length of follow-up (months)**	35 (56)
**Time to last scan (months)**	32 (41)

A total of eight patients who underwent concurrent PVDO/PVR and Chiari decompression were identified. Patient demographics and operative details are included in Table 1. Fifty percent (*n* = 4) of patients had a genetic form of craniosynostosis. Of these, 2 patients had Crouzon syndrome and 2 had multi-suture craniosynostosis, 1 secondary to a CYP26B1 mutation and 1 with an unidentified mutation. Of the remaining 50% (*n* = 4) with non-syndromic craniosynostosis, 2 patients had atypical sagittal synostosis and 2 had multi-suture synostosis. The median age at presentation was 0.4 [1.8] months. All patients had suspected increased ICP pre-operatively; 87.5% (*n* = 7) of patients had Chiari I, while only 1 patient had a Chiari II. The median degree of tonsillar herniation on pre-operative imaging was 10.0 [5.5] millimeters. The degree of herniation ranged from 7.5 mm to 20 mm on pre-operative imaging; 50.0% (*n* = 4) had evidence of syrinx on pre-operative imaging; 62.5% (*n* = 5) of patients were shunt dependent pre-operatively. Seventy five percent (*n* = 6) had undergone prior craniofacial/vault surgery, 50% (*n* = 4) had undergone previous PVDO or PVR, and 25% (*n* = 2) had undergone prior Chiari decompression.

### Surgical outcomes (Tables 3 and 4)

**Table 3:** Operative details and outcomes of eight patients after concurrent posterior vault reconstruction or posterior vault distraction osteogenesis and Chiari decompression. *CF* craniofacial surgery, *PVR* posterior vault reconstruction, *PVDO* posterior vault distraction osteogenesis, *SDH* shunt dependent hydrocephalus

	*N* (%) or median (IQR)
**Age at surgery (years)**	4.0 (4.5)
**Type of posterior vault surgery**	
PVDO	4 (50.0%)
PVR	4 (50.0%)
**Length of stay (days)**	5 (4)
**Distraction distance (mm)**	24 (6)
**Complications**	1 (12.5%)
Distractor infection	1 (12.5%)
**Chiari on post-operative imaging**	
Stable	4 (50.0%)
Improvement	4 (50.0%)
Progression	0 (0.0%)
**Syrinx on post-operative imaging**	
None, unchanged	4 (50.0%)
Resolved	1 (12.5%)
Present, stable	1 (12.5%)
Present, progression	2 (25.0%)
**SDH status**	
Status unchanged, SDH	5 (62.5%)
Status unchanged, no SDH	3 (37.5%)
**Subsequent CF surgery**	4 (50.0%)
Monobloc	2 (25.0%)
Bilateral Fronto-orbital Advancement	1 (12.5%)
Cranioplasty	1 (12.5%)
**Repeat Chiari decompression**	1 (12.5%)
**Improvement in symptoms at first follow-up**	8 (100.0%)
**Improvement in symptoms at last follow-up (*n* = 7)**	6 (85.7%)

**Table 4:** Comparison of clinical outcomes between patients who underwent posterior vault distraction osteogenesis and posterior vault reconstruction. PVR: posterior vault reconstruction. PVDO: posterior vault distraction osteogenesis. CM: Chiari Malformation

*N* (%) or median (IQR)	PVDO	PVR	*p*-value
Radiographic improvement in CM	3/4 (75%)	1/4 (25%)	0.5
Radiographic improvement in Syrinx	0/1 (0%)	1/3 (33%)	1.0
Symptomatic improvement in CM at last follow-up	3/4 (75%)	3/3 (100%)	1.0

Median age at surgery was 4.0 [4.5] years old. Fifty percent (*n* = 4) underwent PVDO and 50% (*n* = 4) underwent PVR. One patient underwent C1 laminectomy during Chiari Decompression (12.5%, *n* = 1). Median length of stay was 5 [4] days. Patients who underwent PVDO had a median distraction distance of 24 [6] millimeters. One patient (12.5%) had a complication after the index procedure, presenting 2.7 months post-distractor placement with concern for an infected distractor and being treated successfully with antibiotics. There were no long-term sequelae.

After concurrent PVDO/PVR and Chiari decompression, 50.0% (*n* = 4) of patients had improvement and 50.0% (*n* = 4) had a stable presentation of CM on post-operative imaging. Median time to most-recent CT or MRI was 32 [41] months. As expected, the patient with a Chiari II malformation demonstrated a stable CM. While most patients with radiologic improvement in their CM were in the PVDO group compared to PVR, this difference was not significant (75% (*n* = 3/4) vs. 25% (*n* = 1/4), *p* = 0.5). We would need a sample size of 20 in each group to reliably detect an effect size of *h* ≥ 1.047 with at least 90% power (probability ≥ 0.9), assuming a two-sided test with a maximum Type I error rate of *α* = 0.05. Chiari status was similar when stratified by syndromic status and age at surgery (*p* > 0.05). Of the four patients who had syrinx on pre-operative imaging, 1 had resolution (25%), 1 was stable (25%), and 2 progressed on post-operative imaging (50%). Syrinx status post-operatively was not significantly different when stratified by operation, syndromic status, or age at surgery (*p* > 0.05).

One hundred percent (*n* = 8) of patients had improvement of symptoms at the first post-operative visit; 85.7% (*n* = 6/7) had improvement of symptoms including headaches, vomiting, somnolence, and signs including fontanelle fullness at the last follow-up. Resolution in clinical symptoms was similar in the PVDO and PVR groups at the last follow-up (75% (*n* = 3/4) vs. 100% (*n* = 3/3), *p* = 1.00). Symptomatic improvement was not significantly different when stratified by syndromic versus non-syndromic craniosynostosis or age at surgery (*p* > 0.05). One patient had symptomatic recurrence at 3 years post-operation, secondary to a worsening syrinx seen on MRI despite stable tonsillar herniation. Ultimately, the patient required two subsequent intradural Chiari decompressions and was the only patient to require repeat decompression post-operatively.

The number of patients who were shunt dependent post-operatively remained the same as pre-operation (62.5%, *n* = 5); 50.0% (*n* = 4) underwent subsequent craniofacial surgery including Monobloc (25%, *n* = 2), bilateral fronto-orbital advancement (12.5%, *n* = 1), and cranioplasty (12.5%, *n* = 1). One patient required repeat Chiari decompression (12.5%, *n* = 1). Median length of follow-up was 35 [56] months.

### Volumetric analyses (Tables 5 and 6)

**Table 5:** Pre- to post-operative volumetric changes among patients who underwent PVDO and PVR. *PVDO* posterior vault distraction osteogenesis, *PVR* posterior vault reconstruction

	PVDO			PVR		
Median (IQR)	Pre-operative	Post-operative	*p*	Pre-operative	Post-operative	*p*
Total volume	1269.1 (114.3)	1480.0 (165.4)	0.03*	1479.4 (116.4)	1574.2 (43.6)	0.6
Anterior vault Volume (cm^3^)	412.3 (105.9)	386.4 (72.7)	0.2	376.3 (69.4)	389.2 (89.6)	0.5
Middle vault volume (cm^3^)	306.7 (52.7)	282.2 (94.8)	0.5	252.5 (22.9)	260.9 (75.0)	1.0
Posterior vault Volume (cm^3^)	562.5 (123.6)	844.1 (351.0)	0.1	884.8 (87.1)	958.4 (41.1)	0.4
Anterior vault ratio	0.32 (0.1)	0.26 (0.06)	0.06	0.26 (0.03)	0.24 (0.06)	0.4
Middle vault ratio	0.23 (0.03)	0.19 (0.09)	1.0	0.17 (0.03)	0.17 (0.04)	0.6
Posterior vault ratio	0.47 (0.10)	0.56 (0.16)	0.2	0.59 (0.01)	0.60 (0.04)	0.2

**Table 6:** Comparison of volumetric changes between patients who underwent PVDO and PVR. *PVDO* posterior vault distraction osteogenesis, *PVR* posterior vault reconstruction

Median (IQR)	PVDO	PVR	*p*-value
Change in total vault volume (cm^3^)	192.9 (56.9)	94.8 (160.0)	0.2
Change in anterior vault volume (cm^3^)	−89.7 (79.4)	−13.1 (33.3)	0.3
Change in middle vault volume (cm^3^)	80.1 (86.7)	8.4 (52.1)	0.5
Change in posterior vault volume (cm^3^)	147.6 (136.6)	124.9 (102.5)	0.2

Seven patients were included in the volumetric analysis. One patient was excluded due to poor pre-operative scan quality. The median time to the most recent CT scan was 25 [34] months. Among patients who underwent PVDO, there was a significant improvement in total vault volume from the pre- to post-operative period (1269.1 [114.3] vs. 1480.0 [165.4] cm^3^, *p* = 0.027). Posterior vault volume improved in both PVDO (562.5 [123.6] vs. 844.1 [351.0] cm^3^, *p* = 0.1) and PVR (884.8 [87.1] vs. 958.4 [41.1] cm^3^, *p* = 0.4) cohorts; however, this did not reach significance. When considering the change in total volume between patients who underwent PVDO and PVR, patients who underwent PVDO tended to have greater improvement in total (192.9 [56.9] vs. 94.8 [160.0], *p* = 0.2) and posterior vault volume (147.6 [136.6] vs. 124.9 [102.5], *p* = 0.2), though this did not reach significance. There was no difference in anterior or middle vault volume between techniques (*p* > 0.05).

## Discussion

The occurrence of CM in patients with syndromic and atypical non-syndromic craniosynostosis is a well-described, but poorly understood phenomenon, and its treatment is an important facet of management protocols [1–3]. In patients with craniosynostosis and concerns for elevated ICP, or syrinx in the setting of CM, posterior fossa decompression is typically employed [4]. These patients may also be recommended to undergo posterior vault expansion and/or remodeling to correct or prevent the sequelae of craniosynostosis [8, 9, 19], with previous scholarship supporting that posterior vault distraction may ameliorate the degree of Chiari I severity [14, 20–22], secondary to increased volumetric expansion [23]. Building on this, we present a series of patients who underwent simultaneous posterior vault surgery and Chiari decompression for the treatment of syndromic and atypical non-syndromic craniosynostosis with CM, focusing on symptoms and radiographic evidence of an alteration in disease state. Our results show that concurrent posterior vault surgery and Chiari decompression is associated with symptomatic improvement at immediate post-operative follow-up, with high rates of longer-term relief (85.7%, *n* = 6/7) at 3 years follow-up. Our complication profile was modest, further supporting a favorable risk/benefit ratio which may provide the foundation for a more rigorous study in the future of the risks and benefits of concomitant surgery.

 Posterior vault surgery, specifically PVDO, is considered a safe and effective operation for the treatment of craniosynostosis, though distractor-related complications are not uncommon [8, 11, 23, 24]. When considering Chiari decompression, Navarro et al. reported a complication rate of 5.6% in patients who underwent extradural decompression compared to 42.1% in patients who underwent durotomy at the time of decompression [5]. Previous literature has corroborated these findings, showing that extradural decompression is safer and less morbid than decompression with duraplasty [25–28]. Further, Cinalli et al. reported no complications post-operatively after concurrent occipital remodeling and extradural decompression [12]. These findings suggest that concurrent posterior vault surgery and extradural Chiari decompression may be a safe and viable option for patients presenting with complex craniosynostosis and CM. The question, which unfortunately remains unanswered by our study, is exactly which group of patients benefits from concurrent surgery and which patients are better served by a staged approach.

In addition to low morbidity, clinical symptoms of symptomatic CM/increased ICP in this series improved after concurrent Chiari decompression and posterior vault surgery. Further, most continued to be symptom-free at extended follow-up (85.7%, *n* = 6/7). Previous studies on posterior vault surgery, specifically PVDO, have demonstrated effective cranial expansion to relieve raised ICP [8, 9, 11, 21, 29], with subjective reports of symptomatic and radiologic improvement post-operatively [8, 21]. McMillan et al. reported a radiological improvement in features of raised ICP in 62% of patients, as evaluated by increased CSF flow or reduction in size of Chiari or syrinx [21]. Existing literature on extradural Chiari decompression has suggested symptomatic resolution rates ranging from 72.2% to 97.2% after extradural Chiari decompression [5, 26]. One patient in our cohort had symptom recurrence three years post-operation and was found to have syrinx progression, despite stable tonsillar herniation, requiring two subsequent intradural Chiari decompressions. Further, another patient in our series, with a Chiari II malformation, had a similarly stable presentation post-operatively. In patients with a high likelihood of substantial arachnoid adhesions, as in the two cases above, this procedure may not be effective. This finding is supported by Navarro et al., who reported 13.5% (*n* = 13) of patients required re-decompression after extradural decompression, three of which were due to new onset hydromyelia or syrinx [5]. Together, these findings suggest that concurrent posterior vault surgery and Chiari decompression are effective for the treatment of symptomatic CM/increased ICP, but that some patients may require more aggressive management in the setting of syrinx development or progression. Further, this technique may be less applicable in patients with known adherent arachnoid adhesions.

On post-operative imaging, 50.0% (*n* = 4) of CMs were improved and 50.0% (*n* = 4) were stable. Interestingly, most patients who had radiologic improvement had undergone PVDO as compared to PVR; however, we are underpowered to detect a statistically significant difference (75% (*n* = 3) vs. 25% (*n* = 1), *p* = 0.5). Previous studies have reported resolution or improvement of CM and syrinx after PVDO, including data from our group that showed 22% (*n* = 2) of patients’ radiologic improvement in CM post-operatively [14]. Preliminary volumetric analyses performed in this study suggest there may be greater improvement in posterior vault volume in patients who underwent PVDO. Previous literature supports this finding, indicating that PVDO is associated with increased posterior volume as compared to conventional vault expansion [19, 30]. Furthermore, Park et al. noted significant increases in intracranial volume and decreased cerebellar tonsillar descent in all patients after complete PVDO to the level of the foramen magnum among patients with craniosynostosis and Chiari I malformation [13]. However, a study by Caldarelli et al. showed that improvements in cerebellar tonsil herniation after bony Chiari decompression may be minimal, despite symptomatic improvement [6]. When considering concurrent occipital vault remodeling and decompression, Cinalli et al. reported similar findings, showing no change in the degree of tonsillar herniation in 50% (*n* = 2) and recurrence in 25% (*n* = 1) of patients [12]. We hypothesize that our findings may be due to the minimally invasive technique utilized during decompression, consisting of foramen magnum widening and occipital dissection only, representing a deviation from the traditional extradural Chiari decompression employing C1 laminectomy [5, 6]. In our study, only one patient underwent C1 laminectomy, with subsequent improvement in CM post-operatively. Overall, previous studies support our findings, reporting a mixed correlation between radiographic reduction of syrinx or tonsillar herniation and outcome or prognosis [5, 31, 32].

Shunt dependent hydrocephalus (SDH) is also frequently encountered in patients with syndromic craniosynostosis, thought to be secondary to venous outflow obstruction and/or constriction of the posterior fossa [33–36]. While hydrocephalus in non-syndromic craniosynostosis is reported to occur at rates similar to the general population [37], it is more common in patients with complex non-syndromic craniosynostosis and tonsillar herniation [33]. Further, CM is a known risk factor for the development of hydrocephalus [36]. In this study, rates of SDH remained constant post-operatively. These findings are similar to a previous study by our group that reported no change in the rate of SDH after PVDO or PVR, though they did not consider concurrent Chiari decompression [14]. Cinalli et al. found that after concurrent occipital remodeling and Chiari decompression, hydrocephalus persisted, requiring VP shunt placement in one patient [12], and suggesting that posterior fossa constriction is only one factor in the development of hydrocephalus in craniosynostosis [36].

While this study provides level three evidence for a combined approach in select patients with craniosynostosis, it is not without significant weaknesses. First, this is a retrospective case series, thus limiting our analysis to the data present in the electronic medical record, which can only conclude correlations and not causation. Further, some patients in our sample had limited follow-up and post-operative scans, specifically a lack of MRIs, which may have limited our radiographic data/conclusions. Our small sample size also limited our ability to assess risk and outcomes with regards to PVDO vs. PVR, as we were underpowered to detect significant differences. Furthermore, the study focuses on a heterogenous group of patients, many of whom share little to no similarity, making it difficult to draw meaningful conclusions. Finally, few of our patients have reached skeletal maturity, and further study of their progress is needed to fully understand the implications of the timing and type of intervention. Despite these limitations, we believe that we have presented a valuable series demonstrating the utility of concurrent PVR/PVDO with Chiari decompression for the treatment of CM in select patients with syndromic and complex non-syndromic craniosynostosis.

## Conclusion

Posterior vault expansion with extradural Chiari decompression in patients with concomitant suspected increased ICP and craniosynostosis appears safe and relatively effective at extended follow-up. Additional study of its long-term effects, patient selection, and comparison of PVDO vs PVR is warranted.

## Data Availability

This data is available upon reasonable request.
